# Tuberous Sclerosis and Bilateral Renal Angiomyolipomas: A Case Report and Literature Review of Emerging Treatment Strategies

**DOI:** 10.1155/2016/4595014

**Published:** 2016-07-25

**Authors:** Francois Jo-Hoy, Omar Tolaymat, Ryan Kunjal, Leighton R. James

**Affiliations:** ^1^Department of Internal Medicine, University of Florida College of Medicine, Jacksonville, FL 32209, USA; ^2^Division of Nephrology and Hypertension, Department of Medicine, University of Florida College of Medicine, Jacksonville, FL 32209, USA

## Abstract

Tuberous sclerosis complex is a rare multisystemic genetic disorder associated with the development of benign hamartomas. Angiomyolipomas are one such characteristic finding that may be seen in 55–80% of tuberous sclerosis complex patients. While being normally asymptomatic, they can also cause significant morbidity and mortality. We present the case of a patient with tuberous sclerosis complex and recently discovered bilateral renal angiomyolipomas, admitted for hematuria who underwent left renal artery embolization; however, worsening renal function necessitated subsequent nephrectomy. Despite still being mainstays of treatment, invasive interventions are now being recommended for specific patient populations as demonstrated in our case. Emerging strategies targeting the PI3K/AKT/mTOR pathway have been shown to reduce the size of angiomyolipomas and are now used to treat asymptomatic cases >3 cm. Our review discusses these treatment options with the intention of increasing awareness of current recommendations and hopefully leading to increased application of these novel therapies that will reduce the need for invasive interventions.

## 1. Introduction

Tuberous sclerosis complex (TSC) is a rare neurocutaneous disorder that is defined by the presence of benign hamartomas [1]. While these are benign tumors, they can have significant clinical manifestations. Angiomyolipomas (AML) are a known entity associated with TSC and are usually asymptomatic but may cause hemorrhage, the risk of which has been associated with tumor size, tumor growth, and presence of aneurysms >0.5 cm [2]. We present a case of a patient with known TSC and AML admitted for abdominal pain and hematuria who required renal artery embolization and eventual nephrectomy. While still being cornerstones of therapy, invasive interventions are now being recommended for specific patient populations as demonstrated by our case. Emerging treatment strategies that target the PI3K/AKT/mTOR pathway are now being utilized and have been shown to decrease AML size and volume.

## 2. Case 

A 45-year-old Caucasian female with a past medical history of TSC presented with a 2-week history of right lower quadrant abdominal pain and gross hematuria. She had been recently seen at another hospital for similar symptoms, had a urine culture positive for* Klebsiella*, and was treated for pyelonephritis with cefdinir. A CT abdomen was also done that demonstrated bilateral AML. The patient reported being diagnosed with TSC as a child but denied any history of seizures or AML. She also denied any family history of TSC. Her abdominal pain was described as severe, “stabbing” in nature, constant, radiating across her abdomen occasionally with no relieving or aggravating factors. The hematuria was noted with every void and was described as “cranberry” colored urine. She denied any other symptoms. On examination she was hemodynamically stable but was found to have angiofibromas involving her face in a butterfly distribution. She also had a firm mass in the right lower quadrant of the abdomen approximately 6.5 cm that was ballotable and which one was able to get above and below on palpation. The abdomen was tender at this site with guarding.

**Figure 1:**
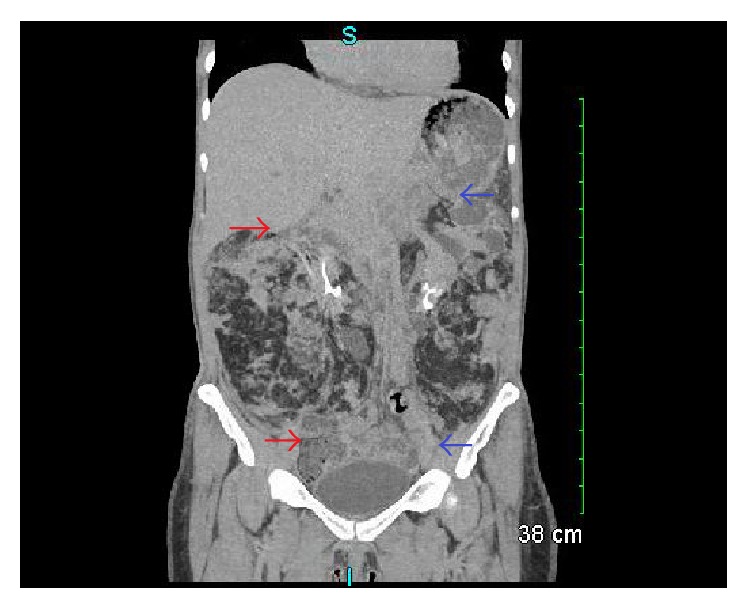
Coronal view of CT urogram showing extensive bilateral AML. Red arrows denote right AML. Blue arrows denote left AML.

**Figure 2:**
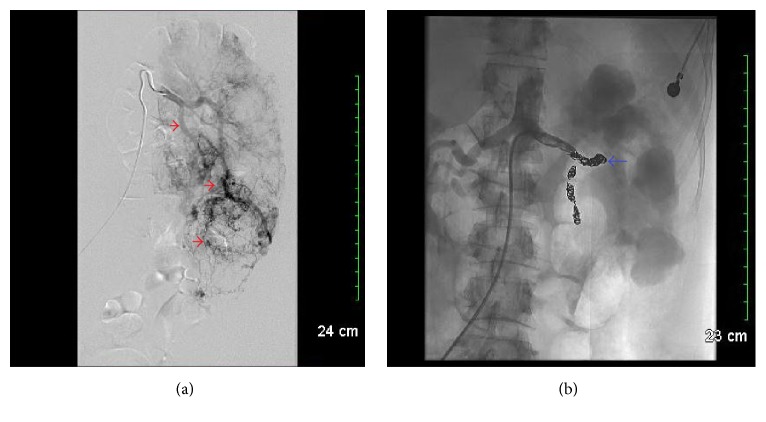
(a) Image of left renal arteriography prior to embolization that showed enlarged kidney with extensive tumor vasculature. Red arrows demonstrate renal vasculature as seen on arteriography. (b) Image after left renal embolization with visualization of metallic coils. Blue arrow indicates metallic coil.

Our patient had a normocytic anemia with a Hb of 10.7 g/dL on admission that remained stable during her stay with a normal coagulation profile (INR 1, PTT 32). UA on admission showed large bilirubin, small ketones, 300 proteins, large WBC, large RBC, and 2 urobilinogens. Initial creatinine was 1.22 mg/dL with eGFR 48 mL/min/1.73 m^2^. A renal and bladder ultrasound was performed in the ED showing echogenic renal parenchyma bilaterally and moderate grade left hydronephrosis. Urology was consulted and a subsequent CT urogram was done demonstrating enlarged bilateral kidneys (right kidney 16 × 12 × 16 cm, left kidney 17 × 11 × 12 cm) and marked heterogeneity with mixed soft tissue and fatty attenuation. The impression was large bilateral renal AML's with marked hydronephrosis, possible areas of extravasated IV contrast within the left kidney, and delayed renal excretion of contrast indicating renal dysfunction (Figure 1). Because no discrete AML could be identified that was amenable to embolization by IR, the patient had cystoscopy with a retrograde pyelogram revealing gross blood effluxed from the left ureter on cystoscopy. Left ureteral hydronephrosis was seen on pyelogram with no clot obstruction, calculus, or external obstruction noted. Ureteroscopy of upper collecting system was done showing bloody urine and lower pole clot, but no bleeding source was identified. The patient was then taken by IR and had renal arteriography from a right groin approach and left renal embolization (Figure 2).

**Figure 3:**
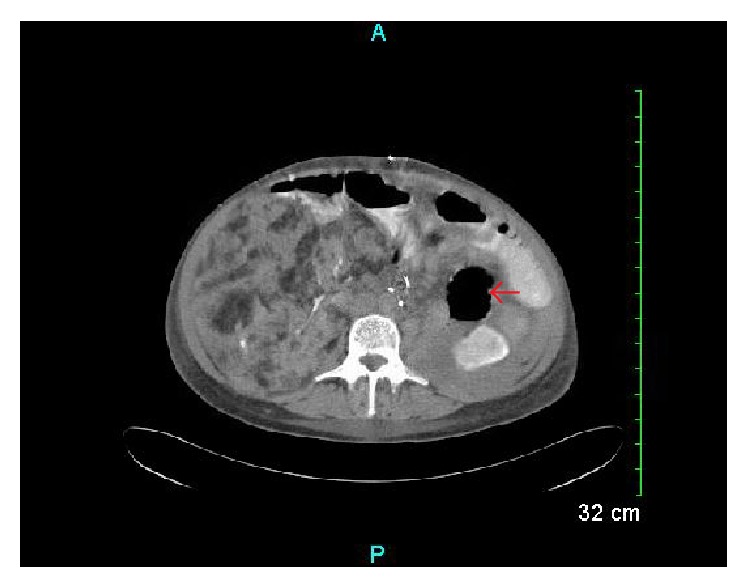
Transverse section of CT abdomen-pelvis after left nephrectomy (indicated by arrow).

Despite embolization, the patient's creatinine and eGFR worsened with creatinine peaking at 2.54 mg/dL (with eGFR 20 mL/min/1.73 m^2^). She also developed hyperkalemia with wide anion gap metabolic acidosis that was treated with bicarbonate IVI. Due to her worsening renal function, embolization was deemed to have been unsuccessful and after consultation with urology and nephrology it was decided that nephrectomy was indicated. Our patient underwent left nephrectomy and left ureterectomy with left adrenal sparing day 5 of admission (Figure 3). The excised kidney was sent to pathology for analysis and was found to measure 30 × 13 × 9 cm and weigh 1850 g. On gross examination the outer surface was tan-gray and nodular. Sections of the kidney were taken that revealed a tan-yellow variegated appearance, beefy red parenchyma, and multiple small cysts (largest measuring 3 cm in diameter). Pathology findings confirmed diagnosis of AML. Creatinine and eGFR improved after surgery (creatinine improved to new baseline of 1.4 mg/dL). Our patient had good convalescence and was discharged with urology and nephrology follow-up but unfortunately has been noncompliant with both.

## 3. Discussion

### 3.1. Tuberous Sclerosis Complex

Tuberous sclerosis complex is a rare genetic disorder characterized by benign hamartomas. It has no sexual or ethnic predilection and its prevalence is thought to be 1 in 6000–9000. It is estimated that at least 2 million people are affected worldwide with 40000 being found in America [5].

TSC can be due to spontaneous mutations or autosomal dominant inheritance with variable expressivity and incomplete penetrance. It has been associated with mutations of the TSC1 gene that codes for hamartin and TSC2 that codes for tuberin. Mutations in these 2 genes are found in 75–80% of those with TSC with TSC2 mutations being more frequent and being associated with a more severe course [1]. Both encoded proteins along with a protein TBC1D7 form the TSC protein complex that suppresses mammalian target of Rapamycin (mTOR), a serine-threonine kinase which is involved in cell proliferation and growth [2, 5].

Associated hamartomas can affect multiple organs and include facial angiofibromas, ungual fibromas, cardiac rhabdomyomas, cortical/subcortical tubers, subependymal nodules, subependymal giant cell astrocytomas, retinal hamartomas, AMLs, pulmonary lymphangioleiomatosis, and renal cell carcinomas (rare). Skin manifestations that are important for diagnosis include “ash leaf spots (hypomelanotic macules)” and Chagrin patches [2]. Radial migration lines and renal cysts can also be seen.

Common presenting symptoms of TSC include cardiac rhabdomyomas which may be seen at 22 weeks of gestation, skin lesions, and seizures. Hypopigmented macules are seen upwards of 90−96% of patients and up to 90% will have seizures [5]. Patients may also develop tuberous sclerosis complex associated neuropsychiatric disorders (TAND) which include autism, ADHD, mood disorders, anxiety disorders, intellectual disability, memory deficits, language deficits, and learning difficulties [6].

Definite diagnosis of TSC is made in the presence of 2 major criteria or 1 major criterion and ≥2 minor criteria. It may also be diagnosed with identification of a pathogenic mutation of the TSC1 or TSC2 gene (that inactivates or prevents protein synthesis of TSC1 or TSC2 proteins or is a missense mutation whose effect on protein function has been established by functional assessment). A possible diagnosis is given if 1 major or ≥2 minor criteria are present.  Table 1 illustrates both major and minor criteria [3]. Our patient was only noted to have 1 major criterion (angiofibromas) and 1 minor criterion (multiple renal cysts) on presentation; however she had already been diagnosed with TSC during childhood and was found to have classic renal AMLs. Due to these findings no further diagnostic workup or imaging was done during admission.

### 3.2. Renal Angiomyolipomas

Renal angiomyolipomas are benign tumors composed of vascular, smooth muscle, and fat tissue. The prevalence of AML has been reported as 0.02–0.1% in males and 0.22–0.29% in females with 20% of affected patients having concomitant TSC [7]. Conversely 55–80% of TSC patients have AML. They are normally asymptomatic but may present with flank or abdominal pain, hematuria, abdominal mass or distension, fever, nausea, vomiting, or progressive loss of renal function due to loss of normal renal parenchyma. It may even result in retroperitoneal hemorrhage (Wunderlich syndrome) that can lead to death [2, 8]. They are often multiple and bilateral with rapid growth in childhood and adolescence and are often associated with micro- and macroaneurysms that predispose patients to hemorrhage. In fact, the vasculatures found within AML are susceptible to rupture as they are thick walled and lack normal elastin. Bleeding risk is associated with tumors >4 cm, tumor growth, and aneurysm >0.5 cm. Despite this risk, CKD and renal failure are the leading cause of mortality in adult patients [2, 8]. It is therefore not surprising that our patient presented with hematuria as she had extensive AML involvement with gross renal enlargement that occupied much of her abdominal cavity.

### 3.3. Renal Angiomyolipoma Treatment

Treatment of renal AML is dependent on its presentation. Until recently the cornerstones of treatment have included selective arterial embolization and nephrectomy. Below we highlight these strategies and also discuss novel therapies that target the PI3K/AKT/mTOR pathway.

### 3.4. Embolization

Selective arterial embolization is the most commonly employed nephron sparing intervention. It can be performed for prophylaxis of high risk AMLs, in the event of acute bleeding, and also prior to nephrectomy to reduce intraoperative bleeding. It is currently recommended that embolization be done in the event of asymptomatic tumors >8 cm or symptomatic tumors that are >4 cm [9]. A systematic review by Murray et al. looked at 524 patients who underwent embolization for renal AML. Of these 93.3% had successful embolization with 20.9% needing repeat intervention. Postembolization syndrome was the most common complication described [7].

### 3.5. Nephrectomy

Both partial and total nephrectomy have been utilized. It offers complete removal of the tumor and allows for histological evaluation. It is also associated with a lower recurrence of disease when compared to embolization but does carry with it an increase in complications [10]. Nephrectomy is now usually reserved for tumors in which embolization would be difficult, if the AML vascular anatomy is complex, if embolization fails, or if malignancy is suspected [7, 9]. In our patient a decline in renal function (measured by increasing creatinine) was noted despite renal artery embolization and was accompanied by metabolic acidosis requiring bicarbonate IVI and hyperkalemia. This prompted consideration of nephrectomy which was subsequently performed. This intervention resulted in improvement of the patient's renal function.

### 3.6. PI3K/AKT/mTOR Pathway Targeted Therapies

The PI3K/AKT/mTOR pathway is well known to play an important role in cell proliferation, angiogenesis, differentiation, and survival. Its dysregulation and activation are seen in many tumors [11]. PI3K (phosphatidylinositol 3 kinase) is a dimer comprising a regulatory and catalytic subunit. When activated it phosphorylates membrane PIP2 (Phosphatidylinositol 4,5-bisphosphonate) and PIP3 (phosphatidylinositol 3,4,5-triphosphate). PIP3 binds and activates PDK1/PDK2 (3-phosphoinositide dependent protein kinases 1 and 2). PDK1 and PDK2 phosphorylate AKT (protein kinase B serine/threonine kinase) which in turn inhibits the TSC complex. The TSC complex naturally inhibits a protein termed Rheb (Ras homolog enriched in brain) that activates mTOR [12]. Mutations involving the TSC complex thus result in overactivation of this pathway.

The importance of this pathway in tumor pathology has led to the development of therapeutic strategies that target specific signaling molecules within the pathway. The Everolimus for angiomyolipoma associated with tuberous sclerosis complex or sporadic lymphangiomyomatosis (EXIST-2) trial was a multicenter, randomized, double blind, placebo-controlled, phase 3 trial of 118 patients with a definite TSC diagnosis and at least one AML ≥3 cm that showed a reduction of AML volume with Everolimus (mTORi) therapy [13]. Favorable outcomes of the EXIST-2 trial led to an open label extension performed by Bissler et al. that looked at long-term efficacy and safety of Everolimus. 118 patients were enrolled with 79 randomized to Everolimus therapy and 39 were given placebo. 112 received Everolimus during the study (33 patients switched from the placebo group). 87.5% of these continued with Everolimus treatment up to the cutoff date of the study. Results of this study showed an improvement in the response rate to treatment when compared to results of the EXIST-2 trial—continued AML volume reduction and no AML related bleeding events. Of those receiving Everolimus therapy, the most commonly reported adverse events (>25% of patients) were nasopharyngitis, stomatitis, headache, acne, hypercholesterolemia, urinary tract infection, and aphthous stomatitis. Most of these were managed by either dose reduction or treatment interruption. Current recommendations therefore advise that asymptomatic AMLs >3 cm are to be treated with mTOR inhibitor in the short term [14]. Our patient had AMLs >3 cm but was not asymptomatic (presented with hematuria and developed worsening renal function) and unfortunately was not a candidate for a Everolimus. This underlines the importance of early AML detection which may have allowed our patient to undergo Everolimus therapy and possibly prevent invasive interventions.

Another mTOR inhibitor, sirolimus (Rapamycin), has also been studied. In a nonrandomized, open label trial involving 25 patients with AMLs and tuberous sclerosis complex or sporadic lymphangioleiomyomatosis performed by Bissler et al., significant reductions in AML volume were observed at 12 and 24 months; however after treatment cessation volumes tended to increase [15]. The multicenter phase 2 nonrandomized open label Trial of Efficacy and Safety of Sirolimus for Treatment of Angiomyolipoma in Tuberous Sclerosis and Sporadic LAM (TESSTAL) by Davies et al. studied 16 patients who were given sirolimus for up to 2 years. Reduction in AML size was again demonstrated with increase in size upon cessation of sirolimus [16].

Other pathway inhibitors are currently being studied and are in various phases of clinical development. These include inhibitors of PI3K, AKT, and other mTOR inhibitors.  Table 2 provides a list of these inhibitors as of 2013 [4]. Despite the many inhibitors being studied, Everolimus is the only pathway inhibitor that has been approved for treatment of AML. The success of these trials is uncovering new treatment options for a potentially debilitating disease process. Implementation of these options will hopefully result in the reduced need for invasive interventions.

## Figures and Tables

**Table 1:** Major and minor diagnostic criteria for tuberous sclerosis complex [3].

Tuberous sclerosis complex diagnostic criteria	
Major criteria	Minor criteria
Angiofibromas (≥3) or forehead plaque	Dental enamel pits (≥3)
Hypomelanotic macules (≥3)	Intraoral fibromas (≥2)
Ungual fibromas (≥2)	Non renal hamartomas
Chagrin patch	Retinal achromatic patch
Multiple retinal hamartomas	Confetti skin lesions
Cortical dysplasias (≥3, including tubers and cerebral white matter radial migration lines)	Multiple renal cysts
Subependymal nodules	
Subependymal giant cell astrocytoma	
Cardiac rhabdomyoma	
Lymphangioleiomyomatosis	

**Table 2:** Table of PI3K, AKT, and mTOR inhibitors in development [4].

PI3K/AKT/mTOR pathway inhibitors		
PI3K	AKT	mTOR
AZD6482	A-443654	AZD-8055
AR245408 (XL 147)	AR-42	BGT226
BAY 80-6946	AR-67 (DB-67)	Everolimus
BKM120	AZD5363	INK-128
BYL719	GSK690693	NVP-BEZ235
GDC-0941	KP372-1	ONC-01910
GDC-0980	MK-2206	OSI-027
GSK2636771	SR13668	PP-242
PF-04691502	Triciribine (API-2)	Rapamycin (sirolimus)
PX-866	VIII	Ridaforolimus
ONC-01910	VQD-002 (API-2)	Temsirolimus
		WYE-354

## References

[1] Samueli S., Abraham K., Dressler A. (2015). Tuberous sclerosis complex: new criteria for diagnostic work-up and management. *Wiener Klinische Wochenschrift*.

[2] De Waele L., Lagae L., Mekahli D. (2014). Tuberous sclerosis complex: the past and the future. *Journal of the International Pediatric Nephrology*.

[3] Northrup H., Krueger D. A. (2013). Tuberous sclerosis complex diagnostic criteria update: recommendations of the 2012 international tuberous sclerosis complex consensus conference. *Pediatric Neurology*.

[4] Owonikoko T. K., Khuri F. R. (2013). Targeting the PI3K/AKT/mTOR pathway: biomarkers of success and tribulation. *American Society of Clinical Oncology Educational Book*.

[5] DiMario F. J., Sahin M., Ebrahimi-Fakhari D. (2015). Tuberous sclerosis complex. *Pediatric Clinics of North America*.

[6] Leclezio L., de Vries P. J. (2015). Advances in the treatment of tuberous sclerosis complex. *Current Opinion in Psychiatry*.

[7] Murray T. E., Doyle F., Lee M. (2015). Transarterial embolization of angiomyolipoma: a systematic review. *Journal of Urology*.

[8] Eijkemans M. J. C., van der Wal W., Reijnders L. J. (2015). Long-term follow-up assessing renal angiomyolipoma treatment patterns, morbidity, and mortality: an observational study in tuberous sclerosis complex patients in the Netherlands. *American Journal of Kidney Diseases*.

[9] Lee S.-Y., Hsu H.-H., Chen Y.-C. (2009). Embolization of renal angiomyolipomas: short-term and long-term outcomes, complications, and tumor shrinkage. *CardioVascular and Interventional Radiology*.

[10] Faddegon S., So A. (2011). Treatment of angiomyolipoma at a tertiary care centre: the decision between surgery and angioembolization. *Journal of the Canadian Urological Association*.

[11] Xia P., Xu X. Y. (2015). PI3K/Akt/mTOR signaling pathway in cancer stem cells: from basic research to clinical application. *American Journal of Cancer Research*.

[12] Giudice F. S., Squarize C. H. (2013). The determinants of head and neck cancer: unmasking the PI3K pathway mutations. *Journal of Carcinogenesis & Mutagenesis*.

[13] Bissler J. J., Kingswood J. C., Radzikowska E. (2013). Everolimus for angiomyolipoma associated with tuberous sclerosis complex or sporadic lymphangioleiomyomatosis (EXIST-2): a multicentre, randomised, double-blind, placebo-controlled trial. *The Lancet*.

[14] Bissler J. J., Kingswood J. C., Radzikowska E. (2016). Everolimus for renal angiomyolipoma in patients with tuberous sclerosis complex or sporadic lymphangioleiomyomatosis: extension of a randomized controlled trial. *Nephrology Dialysis Transplantation*.

[15] Bissler J. J., McCormack F. X., Young L. R. (2008). Sirolimus for angiomyolipoma in tuberous sclerosis complex or lymphangioleiomyomatosis. *The New England Journal of Medicine*.

[16] Davies D. M., De Vries P. J., Johnson S. R. (2011). Sirolimus therapy for angiomyolipoma in tuberous sclerosis and sporadic lymphangioleiomyomatosis: a phase 2 trial. *Clinical Cancer Research*.

